# Sulfur Hexafluoride (SF_6_) versus Perfluoropropane (C_3_F_8_) in the Intraoperative Management of Macular Holes: A Systematic Review and Meta-Analysis

**DOI:** 10.1155/2019/1820850

**Published:** 2019-03-12

**Authors:** Idan Hecht, Michael Mimouni, Eytan Z. Blumenthal, Yoreh Barak

**Affiliations:** ^1^Sackler School of Medicine, Tel-Aviv University, Tel Aviv, Israel; ^2^Department of Ophthalmology, Rambam Health Care Campus, Haifa, Israel; ^3^Ruth and Bruce Rappaport Faculty of Medicine, Technion-Israel Institute of Technology, Haifa, Israel

## Abstract

**Purpose:**

A systematic literature search was conducted to identify and review studies comparing SF_6_ to C_3_F_8_ as a tamponade agent in the intraoperative management of macular holes.

**Methods:**

Publications up to October 2018 that focused on macular hole surgery in terms of primary closure, complications, and clinical outcomes were included. Forest plots were created using a weighted summary of proportion meta-analysis. Analysis was performed separately for SF_6_ and C_3_F_8_. A random effects model was used, and corresponding *I*^2^ heterogeneity estimates were calculated.

**Results:**

Nine pertinent publications studying a total of 4,715 patients were identified in 2000 to 2017, including two randomized studies (*n*=206), two prospective studies (*n*=170), and five retrospective or registry-based studies. Similar rates of closure between SF_6_ and C_3_F_8_ were reported in eight out of nine studies, regardless of subgroup analyses. All studies reporting visual outcomes showed similar results when comparing SF_6_ to C_3_F_8_ at one to six months of follow-up. Neither agent was clearly associated with increased risk of ocular hypertension, cataract formation, or other adverse events. Meta-analytic pooling of the closure rates in the SF_6_ group resulted in 91.73% (95% confidence interval: 88.40 to 94.55, *I*^2^: 38.03%), and for C_3_F_8_, the closure rate was 88.36% (95% confidence interval: 85.88 to 90.63, *I*^2^: 0.0%).

**Conclusions:**

Both SF_6_ and C_3_F_8_ appear to have achieved similar visual outcomes and primary closure rates and neither was associated with an increased risk of adverse events. Considering the more rapid visual recovery with SF_6_, there appears to be no evidence to support C_3_F_8_ as the tamponade agent of choice for macular hole surgery.

## 1. Introduction

Macular hole surgery was first described by Kelly and Wendel in 1991 [1]. Although their 5-step technique has remained largely unchanged, debate still exists concerning several key aspects of the procedure [2]. These include whether to peel the internal limiting membrane (ILM), the use and type of dye, and the duration of face-down positioning following surgery [3, 4].

Another heatedly debated aspect is the choice of tamponading agent [3, 5, 6]. The most commonly used compounds are sulfur hexafluoride (SF_6_) and perfluoropropane (C_3_F_8_). However, hexafluoroethane (C_2_F_6_), room air, and silicone oil are also employed [7]. Although the procedure was initially described with use of SF_6_, subsequent clinical trials used C_3_F_8_ further adding to the debate [1, 8–10]. To the authors' best knowledge, no systematic review or meta-analysis has yet been published, comparing the two agents.

The aim of this study was to systematically review the literature for studies that compared SF_6_ to C_3_F_8_ as a tamponade agent in the intraoperative management of macular hole surgery, perform a pooled meta-analysis of the data, and discuss any differences found with respect to clinical outcomes and adverse effects.

## 2. Materials and Methods

### 2.1. Literature Search

**Figure 1:**
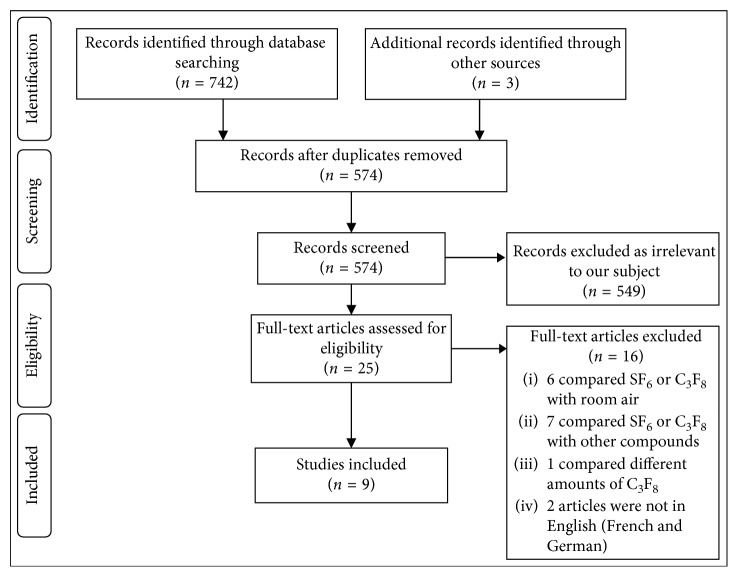
Flow diagram of the inclusion process. Flow diagram of the search and inclusion process based on the Preferred Reporting Items for Systematic Reviews and Meta-Analyses (PRISMA) guidelines [11].

A systematic literature search was conducted using MEDLINE, Scopus, Google Scholar, and The Cochrane Library, reviewing all available published clinical studies up to October 2018. The following keywords were used, in various combinations: macular hole, MH, sulfurhexafluoride, sulfur hexafluoride, SF_6_, perfluoropropane, C_3_F_8_, gas, short-acting, long-acting, tamponade, bubble, pneumatic, and retinopexy. A bibliographic search of relevant studies identified additional publications. A flow diagram of the screening and inclusion process is illustrated in Figure 1.

### 2.2. Eligibility Criteria

Full publications in English that directly compare SF_6_ and C_3_F_8_ in the intraoperative management of macular hole surgery, in terms of primary closure, complication rates, and clinical outcomes were included (not abstracts or letters to the editor). Studies performed on animal or cadaver eyes, as well as case reports and nonempirical opinion articles, were excluded.

### 2.3. Screening and Synthesis

The review process was conducted under the guidance of the PRISMA (Preferred Reporting Items for Systematic Reviews and Meta-Analyses) criteria to support reporting [11]. Two reviewers (IH and MM) independently implemented the search strategy for relevant publications. Selected publications were then approved by the senior investigator who also devised the search strategy (YB).

### 2.4. Statistical Analyses

To better illustrate the main anatomical outcome of primary closure, forest plots were created using a weighted summary of proportion meta-analysis. Analysis was performed separately for SF_6_ and C_3_F_8_. A random effects model was used (as implemented by DerSimonian and Laird, 1986 [12]), and corresponding *I*^2^ heterogeneity estimates were generated. Data were tabulated and analyzed using SPSS for Windows version 22.0 software by IBM Inc. (Armonk, NY, USA). Graphs were created using MedCalc software version 16 (Mariakerke, Belgium).

## 3. Results

Data comparing the use of SF_6_ and C_3_F_8_ for macular hole surgery were extracted from nine studies published during 2000 to 2017 (Table 1), studying a total of 4,715 patients. Two of the studies were randomized by design and included a total of 206 patients. Two studies prospectively enrolled patients without randomization (170 patients), and the remaining five were retrospective or registry based. Figure 1 illustrates the flow of the inclusion process.

### 3.1. Intraoperative Management

Several differences existed between the studies with respect to the surgical technique. The first relates to ILM removal as a routine part of the operation.

ILM peeling was routinely performed in six of the nine studies. In two studies (Essex et al. [4] and Tognetto et al. [7]), a majority of patients (97.7% and 67.7%, respectively) underwent ILM peeling while the remainder did not. The authors elected not to routinely perform ILM peeling of prospectively enrolled patients in only one study (Mulhern et al.) [16].

Tognetto et al. expanded their investigation, comparing the outcomes of patients who underwent ILM peeling with those who had not [7]. They analyzed the retrospective data of 1,627 patients from different countries, who had been operated on for idiopathic macular hole. They found that ILM peeling improved hole closure only for longstanding macular holes or those in advanced stages [7].

A second aspect in which studies differed was the choice of gas concentration. The SF_6_ concentration was 20% in four of the nine studies. Mulhern et al. used 23%, Kumar et al. used 25%, and Briand et al. used between 20 and 25% SF_6_. The C_3_F_8_ concentrations similarly varied, ranging from 13% used by Modi et al. to 18% by Kumar et al. However, most used concentrations ranged between 14% and 16% C_3_F_8_ (Table 1).

### 3.2. Postoperative Management

Face-down positioning varied between studies. Most studies recommended two to seven days of prone positioning; however, some (e.g., Mulhern et al.) requested patients who received SF_6_ maintain a prone head position for up to 4 weeks [16].

A registry-based analysis by Essex et al. sheds further light on this topic [4]. They analyzed the outcome of 2,367 patients from Australia and New Zealand who underwent primary idiopathic macular hole surgery. Approximately 26% of these patients were not advised to remain in prone position postoperatively, and among those who were advised, most were instructed to remain prone between three and seven days. Essex et al. further compared the outcomes between patients who were not advised a postoperative face-down position to those who were and found that no prone positioning was noninferior for holes of less than 400 *μ*m in diameter, while for larger holes noninferiority could not be concluded [4].

### 3.3. Primary Anatomical Closure

Most studies used anatomical closure as the primary outcome; their results are summarized in Table 1. Closure rates ranged from 85% to 100%, with most in the 90% to 95% range.

A comparison of SF_6_ and C_3_F_8_ showed similar rates of closure in eight of the nine studies, including the two randomized controlled studies by Casini et al. [3, 5]. One study by Essex et al. using a more stringent noninferiority analysis with a relatively narrow noninferiority margin (5%) also demonstrated noninferiority of SF_6_ compared to C_3_F_8_ [4]. Only one study by Tognetto et al. reported a higher success rate with SF_6_ (93.6% versus 87.2%); however, the authors provide no statistical foundation for this claim [7].

Several studies further analyzed subgroups in an attempt to detect a subset of patients, which might benefit from one agent over the other. Modi et al. examined different hole sizes, stages, and durations; Casini et al. also examined different stages; and Kim et al. compared the agents independently for different stages, durations of symptoms, presence of posterior vitreous detachment, and whether indocyanine green dye was used for ILM peeling [3, 6, 15]. They all found SF_6_ to produce results similar to C_3_F_8_ regardless of the subgroup analyzed (Table 1).

### 3.4. Visual Acuity

Six studies reported visual acuity outcomes following surgery [3, 5, 6, 14–16]. All showed similar results when comparing SF_6_ to C_3_F_8_ at one to six months of follow-up (Table 1). Improvement following SF_6_ gas ranged from 2.7 to 5.9 Early Treatment Diabetic Retinopathy Study (ETDRS) lines, while following use of C_3_F_8_ ranged between 2.7 and 6.9 ETDRS lines. Five of the six studies report comparative statistics on visual improvement between groups, and all find no significant differences (Table 1). The randomized trial by Casini et al. as well as the prospective cohort study by Xirou et al. showed that visual acuity was initially better in the group treated with SF_6_ but was eventually found to be similar [3, 14].

### 3.5. Complications

Most studies reported on short-term and long-term complication rates in each group. The randomized controlled trial by Briand et al. showed similar results in terms of cataract development and extraction, a similar time interval between surgery and cataract extraction (*p*=0.184) and similar rates of retinal tears (4% vs. 7%), retinal detachment (4% vs. 0%), and ocular hypertension (21% vs. 19%, all *p*=1.0) [5]. The retrospective report by Modi et al. on 177 patients revealed a decreased incidence of cataract and ocular hypertension (1.99 vs. 4.02 mmHg) in the group treated with SF_6_, as well as a nonsignificantly lower incidence of glaucoma (9.0% vs 6.1%) [6]. Xirou et al., in a prospective study on 46 patients, revealed similar elevations of intraocular pressure [14]. Kim et al. reported similar rates of cataract development; however, myopic shift rates were greater in the C_3_F_8_ group (SF6: −0.82 diopters vs. C_3_F_8_: −1.42 diopters for phakic patients, *p*=0.016). Finally, in the prospective trial by Mulhern et al., similar rates of posterior subcapsular cataract were seen (55% after C_3_F_8_ vs. 37% after SF_6_; *p*=0.20) and mean intraocular pressure spike maximum was nonsignificantly higher in the SF_6_ group (32.5 mmHg vs. 23.7 mmHg; *p*=0.131) [16].

### 3.6. Pooled Analysis of Primary Closure

**Figure 2:**
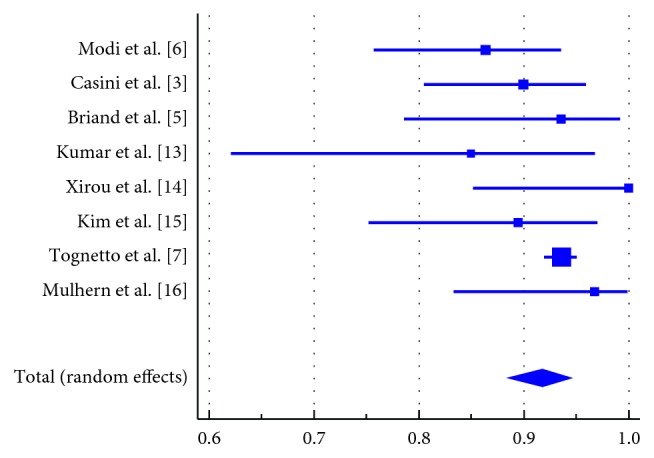
Forest plot of the proportion of patients who achieved anatomical closure following the use of SF_6_ gas. Meta-analytic pooling of the proportion of patients who achieved anatomical closure following surgery in the SF_6_ group. Pooled estimate was 91.73% (95% confidence interval: 88.40% to 94.55%, *I*^2^: 38.03%, *p* value for heterogeneity = 0.126). Size of the squares is proportional to the number of cases in the study. Error bars represent 95% confidence intervals. The diamond shape represents the pooled estimate.

**Figure 3:**
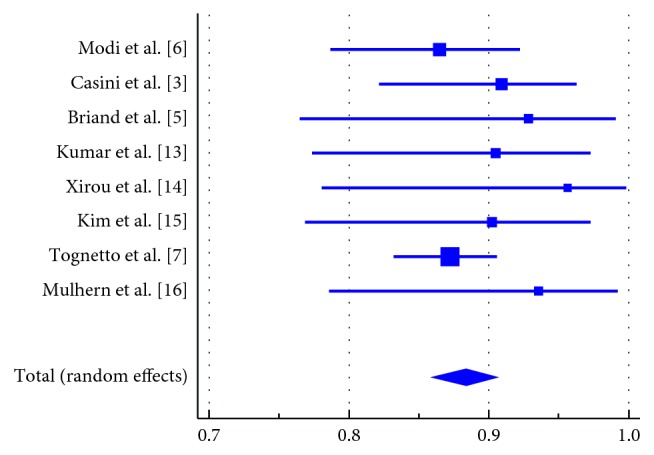
Forest plot of the proportion of patients who achieved anatomical closure following the use of C_3_F_8_ gas. Meta-analytic pooling of the proportion of patients who achieved anatomical closure following surgery with C_3_F_8_: pooled estimate was 88.36% (95% confidence interval: 85.88% to 90.63%, *I*^2^: 0.0%, *p* value for heterogeneity = 0.864); size of the squares is proportional to the number of cases in the study. Error bars represent 95% confidence intervals. The diamond shape represents the pooled estimate.

Forest plots of proportion of patients who achieved anatomical closure with each compound are presented in Figures 2 and 3. Meta-analytic pooling of the closure rates in the SF_6_ group resulted in 91.73% (95% confidence interval: 88.40 to 94.55, *I*^2^: 38.03%, *p* value for heterogeneity = 0.126). For the patients who received C_3_F_8_, the pooled anatomical closure rate was 88.36% (95% confidence interval: 85.88 to 90.63, *I*^2^: 0.0%, *p* value for heterogeneity = 0.864).

## 4. Discussion

In this study, a systematic review and meta-analysis of studies comparing SF_6_ and C_3_F_8_ gas tamponades for macular hole surgery was performed. Macular hole closure rates were found to be similar regardless of whether SF_6_ or C_3_F_8_ was used and were typically in the 90–95% range. Visual acuity outcomes were also similar but tended to improve faster with SF_6_. Rates of complications (including cataract formation, ocular hypertension, and retinal tears) varied among studies and appeared to be inconsistently related to either agent.

Following macular hole repair, contact of the gas bubble with the retina causes the extrusion of subretinal fluid and maintains anatomical position, as well as possibly providing a scaffold for cellular proliferation [14]. It therefore seems reasonable that a larger, longer-acting, bubble would provide further benefit. However, some evidence suggests that hole closure occurs very early in the postoperative period, perhaps as early as the first 24 hours [17]. A recent study by Masuyama et al. showed that repair of the macular hole typically occurs between 4 and 7 days postoperatively and is presumed to be facilitated by ganglion and Muller cells [18]. Long-lasting gasses (such as C_3_F_8_) may offer more extensive tamponade; however, they also impair vision longer. It is possible that such longer-acting agents are not required, considering the short timescales in which macular hole repair occurs. The outcomes of this review appear to support this notion, as use of SF_6_ produced similar clinical outcomes to C_3_F_8_ in terms of both primary closure and visual outcomes.

This study has several limitations. It included nonrandomized trials in the analysis. However, given the small number of randomized studies performing a direct comparison of SF_6_ and C_3_F_8_ for macular hole surgery, we elected to include relevant retrospective and registry-based studies, in order to increase the power of the meta-analysis. Given the findings of this study, the need for more prospective randomized studies evaluating this comparison may be questionable. In addition, several differences between the studies concerning the surgical technique may have introduced biases to the analyses. However, we discussed and compared variations in surgical technique found across these studies.

## 5. Conclusions

To the authors' best knowledge, this is the first systematic review and meta-analysis comparing SF_6_ and C_3_F_8_ as a tamponading agent for macular hole surgery. We found that SF_6_ and C_3_F_8_ resulted in both similar visual outcomes as well as similar primary closure rates. Neither agent was clearly associated with increased risk of ocular hypertension, cataract formation, or other adverse events. Visual recovery with SF_6_ tended to occur earlier. It is probable that shorter-acting tamponade agents such as SF_6_ may be sufficient for macular hole surgery; we found no evidence to support C_3_F_8_ as the tamponade gas of choice.

## Figures and Tables

**Table 1:** Publications reporting on clinical outcomes of macular hole repair using either SF_6_ or C_3_F_8_ gas tamponade.

Study [Ref]	Year	Design	Number of patients	Postoperative face-down positioning duration	Gas concentration	ILM peeling	Anatomical closure	Results: primary closure	Results: visual improvement^*∗*^	Results: complications
Modi et al. [6]	2017	Retrospective comparative study	SF_6_: 67 C_3_F_8_: 111Total: 177	SF_6_: 45 minutes of every hour (duration NR) C_3_F_8_: 45 minutes of every hour (duration NR)	20% SF_6_ 13% C_3_F_8_	All cases	SF_6_: 86.4% C_3_F_8_: 86.5%	Similar (*p*=0.982), irrespective of hole size, stage, or duration	SF_6_: 2.7 lines (0.900 ± 0.383 to 0.629 ± 0.375 logMAR) at 3–6 months C_3_F_8_: 4.2 lines (1.03 ± 0.39 to 0.61 ± 0.40 logMAR) at 3–6 months No significant difference (*p*=0.066)	SF_6_ exhibited a decreased incidence of cataract and ocular hypertension (1.99 vs. 4.02 mmHg) as well as a nonsignificantly lower incidence of glaucoma (9.0% vs. 6.1%)
Casini et al. [3]	2016	Randomized controlled trial	SF_6_: 70 C_3_F_8_: 77 Total: 147	SF_6_: 2 days C_3_F_8_: 2 days	20% SF_6_ 14% C_3_F_8_	All cases	SF_6_: 90% C_3_F_8_: 91%	Similar, independent of stage	SF_6_: 5.9 lines (0.894 to 0.301 logMAR) at 1 month C_3_F_8_: 6.9 lines (0.965 to 0.272 logMAR) at 1 month	Patients treated with SF_6_ experienced greater improvement of visual acuity at 1 week postoperatively (*p* < 0.01) but not at 1 month
Essex et al. [4]	2016	Registry-based study	SF_6_: 1,653 C_3_F_8_: 702 Total: 2,456	SF_6_: 0–14 days C_3_F_8_: 0–14 days	NR	97.7% of cases	Mean of both 95.0% (individually NR)	SF_6_ noninferior, regardless of macular hole size	NR	NR
Briand et al. [5]	2015	Randomized controlled trial	SF_6_: 31 C_3_F_8_: 28 Total: 59	SF_6_: 7 days C_3_F_8_: 14 days	20–25% SF_6_ 15% C_3_F_8_	All cases	SF_6_: 93.3% C_3_F_8_: 92.9%	Similar (*p*=0.943)	SF_6_: 3.5 lines at 12 months C_3_F_8_: 3.4 lines at 12 months No significant difference (*p*=0.787)	Similar in terms of cataract development and extraction and adverse events
Kumar et al. [13]	2014	Prospective cohort study	SF_6_: 20 C_3_F_8_: 42 Total: 62	SF_6_: 18 hours daily for 3 days C_3_F_8_: 18 hours daily for 3 days	25% SF_6_ 18% C_3_F_8_	All cases	SF_6_: 85% C_3_F_8_: 90.5%	Similar (*p*=0.67)	NR	NR
Xirou et al. [14]	2012	Prospective cohort study	SF_6_: 23 C_3_F_8_: 23 Total: 46	SF_6_: 2 days C_3_F_8_: 2 days	20% SF_6_ 14% C_3_F_8_	All cases	SF_6_: 100% C_3_F_8_: 96%	Similar	SF_6_: 3.8 lines (0.67 ± 0.20 to 0.29 ± 0.12 logMAR) at 6 months C_3_F_8_: 2.8 lines (0.90 ± 0.10 to 0.62 ± 0.23 logMAR) at 6 months No significant difference (*p*=0.06)	Patients treated with SF_6_ experienced greater improvement of visual acuity initially but was similar in the following 6 months. Similar elevations of intraocular pressure
Kim et al. [15]	2008	Retrospective comparative study	SF_6_: 38 C_3_F_8_: 41 Total: 79	SF_6_: 7 days C_3_F_8_: 7 days	20% SF_6_ 16% C_3_F_8_	All cases	SF_6_: 90% C_3_F_8_: 91%	Similar (*p*=0.91), regardless of stage, duration of symptoms, presence of PVD, and use of ICG dye for ILM peeling	SF_6_: 4.9 lines (0.86 ± 0.41 to 0.37 ± 0.43 logMAR) at 12 months C_3_F_8_: 5.4 lines (0.99 ± 0.43 to 0.45 ± 0.35 logMAR) at 12 months No significant difference (*p* > 0.30)	Cataract development was similar. Myopic shift was greater in the C_3_F_8_ group (*p*=0.016)
Tognetto et al. [7]	2006	Retrospective cohort study	SF_6_: 1,004 C_3_F_8_: 337 Total: 1,627	SF_6_: NR C_3_F_8_: NR	NR	67.7% of cases	SF_6_: 93.6% C_3_F_8_: 87.2%	Higher success rates with SF_6_	NR	NR
Mulhern et al. [16]	2000	Prospective comparative study	SF_6_: 31 C_3_F_8_: 31 Total: 62	SF_6_: 14–30 days C_3_F_8_: 6 days	23% SF_6_ 16% C_3_F_8_	Not routinely performed	SF_6_: 96.7% C_3_F_8_: 93.5%	Similar (*p*=1.0)	SF_6_: 2.8 lines (0.86 ± 0.18 to 0.575 ± 0.31 logMAR) at 3 months C_3_F_8_: 2.7 lines (0.88 ± 0.21 to 0.61 ± 0.37 logMAR) at 3 months No significant difference (*p*=0.719)	Incidence of posterior subcapsular cataract was similar. Mean IOP spike maximum was nonsignificantly higher in the SF_6_ group

NR, not reported; C_3_F_8_, perfluoropropane; SF_6_, sulfur hexafluoride; ILM, internal limiting membrane; PVD, posterior vitreous detachment; ICG, indocyanine green; IOP, intraocular pressure. ^*∗*^Expressed in Early Treatment Diabetic Retinopathy Study (ETDRS) chart lines.
